# Differential Coupling of Self-Renewal Signaling Pathways in Murine Induced Pluripotent Stem Cells

**DOI:** 10.1371/journal.pone.0030234

**Published:** 2012-01-23

**Authors:** Luca Orlando, Yolanda Sanchez-Ripoll, James Foster, Heather Bone, Claudia Giachino, Melanie J. Welham

**Affiliations:** 1 Centre for Regenerative Medicine, Department of Pharmacy and Pharmacology, University of Bath, Bath, United Kingdom; 2 Department of Clinical and Biological Sciences, University of Turin, Orbassano (Torino), Italy; Universitätsklinikum Carl Gustav Carus an der Technischen Universität Dresden, Germany

## Abstract

The ability to reprogram somatic cells to induced pluripotent stem cells (iPSCs), exhibiting properties similar to those of embryonic stem cells (ESCs), has attracted much attention, with many studies focused on improving efficiency of derivation and unraveling the mechanisms of reprogramming. Despite this widespread interest, our knowledge of the molecular signaling pathways that are active in iPSCs and that play a role in controlling their fate have not been studied in detail. To address this shortfall, we have characterized the influence of different signals on the behavior of a model mouse iPSC line. We demonstrate significant responses of this iPSC line to the presence of serum, which leads to profoundly enhanced proliferation and, depending on the medium used, a reduction in the capacity of the iPSCs to self-renew. Surprisingly, this iPSC line was less sensitive to withdrawal of LIF compared to ESCs, exemplified by maintenance of expression of a Nanog-GFP reporter and enhanced self-renewal in the absence of LIF. While inhibition of phosphoinositide-3 kinase (PI3K) signaling decreased iPSC self-renewal, inhibition of Gsk-3 promoted it, even in the absence of LIF. High passages of this iPSC line displayed altered characteristics, including genetic instability and a reduced ability to self-renew. However, this second feature could be restored upon inhibition of Gsk-3. Collectively, our data suggest modulation of Gsk-3 activity plays a key role in the control of iPSC fate. We propose that more careful consideration should be given to characterization of the molecular pathways that control the fate of different iPSC lines, since perturbations from those observed in naïve pluripotent ESCs could render iPSCs and their derivatives susceptible to aberrant and potentially undesirable behaviors.

## Introduction

Induced pluripotent stem cells (iPSCs) are somatic cells reprogrammed to pluripotency by the over-expression of specific sets of genes. Mouse iPSCs were first generated by introducing the combination of Oct3/4, Sox2, Klf4 and c-Myc [1] and they have now been obtained using many approaches [2]. Since the discovery of iPSCs, the main goal of researchers has been to obtain them with increased efficiency and using techniques that could allow for their use in clinical applications. Many initial studies focused on the similarities between embryonic stem cells (ESCs) and iPSCs, including assessment of pluripotency by testing for their ability to contribute to formation of chimeras (germline transmission) [3]–[7], as well as assessing histone modifications [8] and methylation patterns [9]. In spite of significant technical improvements in the ability to achieve reprogramming, as well as in understanding the biological mechanisms underlying iPSC generation, an in-depth analysis of the fine molecular regulation of iPSC fate and the response of iPSCs to different stimuli is still lacking in literature. In fact, despite the similarities in morphology and the ability to pass the most stringent test of pluripotency (germline transmission), it has become apparent more recently that iPSCs exhibit some important differences when compared to ESCs, exemplified by major differences in mRNA and miRNA expression profiles [10]–[12]. Moreover, the starting conditions of reprogramming appear to influence the behavior of iPSC lines generated [13]–[15] and recently iPSC lines have been shown to retain a transcriptional and epigenetic ‘memory’ of the differentiated cells from which they were derived [16], [17]. This raises the prospect that iPSC lines may respond to a different repertoire of signals, compared to pluripotent ESCs, that is, at least in part, dictated by their cellular origin.

Several extrinsic factors, signaling pathways and transcription factors are known to play important roles in controlling self-renewal and pluripotency of mouse ESCs. The transcription factors include those used to generate iPSCs, the most important being Oct4, Sox2 and Nanog [2]. Of the extrinsic factors, leukemia inhibitory factor (LIF) plays a key role through activation of the Signal transducer and activator of transcription factor 3 (Stat-3) and c-Myc [18]–[20]. Bone morphogenetic proteins 2 and 4 (BMP2/4), present in serum or when added exogenously to serum-free media, cooperate with LIF to promote self-renewal by inducing expression of Inhibitor of Differentiation, Id2 [21]. Although LIF also activates the extracellular-regulated kinases Erk1 and Erk2, the activity of these Mitogen-activated protein (MAP) kinases opposes, rather than promotes pluripotency [22] and in serum-free conditions it has been demonstrated that inhibition of Erk1 and 2, in addition to inhibition of glycogen synthase kinase 3 (Gsk-3), is sufficient to maintain mouse ESCs in a pluripotent ‘ground’ state [23]. Other studies have also reported that inhibition of Gsk-3 promotes self-renewal [24], [25], via β-catenin-dependent and independent mechanisms [26]. Activation of the Phosphoinositide-3 kinase (PI3K) pathway has been associated with controlling proliferation of ESCs [27]–[30], with mTOR playing a key role [29]. PI3K-dependent signaling has also been shown to play a role in optimal maintenance of mouse ESC self-renewal [31]–[34], at least in part through PI3K-mediated inhibition of Gsk-3 activity [35].

In this study, we investigated whether the fate of mouse iPSCs is controlled via similar mechanisms to those established in mouse ESCs. Activation of LIF-Stat-3 and PI3K signaling appear comparable between iPSCs and ESCs. However, we demonstrate that the model iPSC line we have studied, which was derived from fibroblasts [3], exhibits enhanced responsiveness to serum-containing factors, reduced sensitivity to LIF withdrawal and increased responsiveness to inhibition of Gsk-3. Furthermore, activation of Erk1 and 2 is impaired in this iPSC line. We also report that following extended passage this model iPSC line exhibits an altered karyotype, indicative of genomic instability, which correlates with altered behavior. The intriguing possibility that in addition to transcriptional and epigenetic memory the signaling pathways controlling the fate of iPSCs may also be influenced by their cell of origin is discussed on the basis of our results.

## Results

### Dependency of murine iPSC fate on culture conditions

There have been few studies examining the influence of extracellular factors on and the molecular signaling pathways involved in the regulation of cell fate of iPSC lines. Therefore, we have undertaken a study, using one of the earliest murine iPSC lines generated as our model [3], to characterize both the influence of different stimuli on the fate of this line and compare these responses to those of a widely used murine ESC line, namely E14tg2a [36]. Importantly, the iPSC line selected has been demonstrated to be pluripotent and able to give rise to chimeras [3]. Both lines express GFP under the control of the endogenous Nanog promoter, providing a facile means of assessing cell fate.

**Figure 1:**
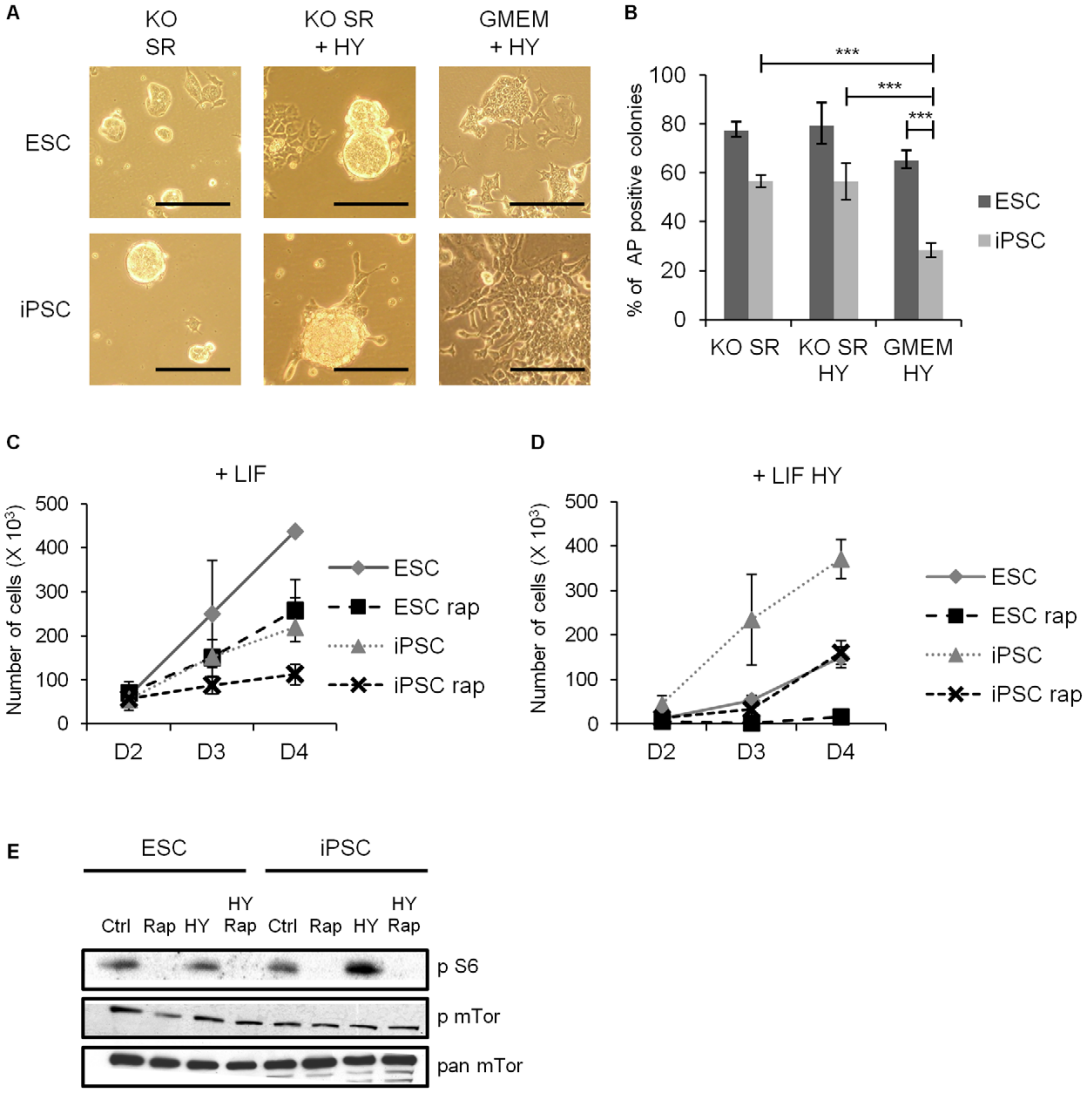
Culture conditions influence iPSC self-renewal and proliferation. **A** ESCs or iPSCs (passage 21) were plated onto gelatin-coated dishes in KnockOut-DMEM (KO) supplemented with KO Serum Replacement (SR) and LIF in the presence or absence of 10%(v/v) Hyclone serum (HY) or GMEM supplemented with LIF and 10% (v/v) HY, as indicated. 48 hours after plating cultures were observed by light microscopy and photographs taken. Representative images are shown, scale bar = 200 µm. **B** The self-renewal capacity of ESCs and iPSCs were evaluated by alkaline phosphatase (AP) expression in clonal assays following 4 days culture in different conditions described in **A**. The average percentage of AP positive colonies ± SEM are shown from three independent experiments. Two-tailed paired t-tests indicate the following significance *** = p<0.005. **C** and **D** Cells where plated at day 0 in KO SR+LIF (**C**) or the same media supplemented with 10% (v/v) HY (**D**). Rapamycin (5 nM) or DMSO (1∶10000, as control) were added 24 hours after seeding, and kept in the medium for the duration of the experiment. Cells were harvested after 48 (D2) 72 (D3) and 96 (D4) hours and counted in triplicate. The average values ± SEM are shown from three independent experiments. **E** ESCs or iPSCs were seeded in KO SR+LIF at 8000 cells/cm^2^. After 24 h DMSO 1∶10000 (ctrl), 5 nM Rapamycin (Rap), 10% (v/v) Hyclone serum (HY) or serum and rapamycin together (HY Rap) were added to the cultures. After 24 h treatment protein extracts were prepared, separated through 10% acrylamide gels using SDS PAGE and immunoblotted using the antibodies indicated.

The extracellular environment can influence the ability of ESCs to self-renew and retain pluripotency and so we first examined the effect of different culture conditions on the growth and fate of our model iPSC line in comparison to murine ESCs. As the iPSC line was originally cultured on a feeder layer of mouse embryo fibroblasts (MEFs), we investigated the effect of culturing this iPSC line on gelatin-coated plates, instead of MEFs, in different media. As shown in Figure 1A, when cultured in KnockOut-DMEM (KO) supplemented with KO Serum Replacement (SR) and LIF, the iPSC line exhibited a morphology typical of that observed with ESCs, with compact, phase-bright colonies predominating. When KO medium was supplemented with serum (we retained SR supplementation in this medium so that we could assess the effect of different treatments using the same basal conditions) the morphology of both ESCs and iPSCs were similar, with a mixture of compact and flattened colonies typically observed. However, when cultured in GMEM plus 10% Hyclone serum (HY) the morphology of iPSCs quickly changed such that they had a more flattened appearance, resembling cells with a more differentiated phenotype. ESCs cultured in the same conditions exhibited a more flattened morphology, but still grew tightly packed within colonies. To test whether iPSCs and ESCs cultured under these different conditions retained the capacity to self-renew, we conducted clonal assays, based on alkaline phosphatase staining, which have been widely used to assess the capacity of ESC populations to retain their pluritpotency. Alkaline phosphatase is expressed only by undifferentiated ESCs and its expression is rapidly lost upon differentiation, hence these assays provide a convenient way to measure self-renewal. iPSCs cultured in GMEM plus serum and LIF exhibited a greatly reduced ability to self-renew, compared with the same cells cultured in KO SR plus LIF (Fig. 1B). This effect seems not to be directly correlated to the presence of serum, but rather the combination of GMEM and serum, as when 10% serum was added to KO SR medium iPSCs retained a morphology similar to ESCs (Fig. 1A) and their capacity to self-renew was similar to that observed when cultured in KO SR plus LIF without serum (Fig. 1B).

We also investigated the growth of iPSCs and ESCs in different media. iPSCs clearly grew more slowly in KO SR plus LIF than ESCs, with a doubling time of approximately 24 hours against 17 hours for ESCs (Fig. 1C). We did not observe any increase in the number of non-viable cells in iPSC cultures compared to ESC cultures, so we consider it unlikely that this decrease in growth is due to increased apoptosis. The data shown in Fig. 1B also suggest that differentiation is not increased in iPSCs under these conditions, so this also seems unlikely to contribute to the decreased growth observed. Interestingly, when serum was included, proliferation of iPSCs increased considerably, such that their growth was more rapid than that of ESCs, with a doubling time of about 13 hours against 15 hours for ESCs (Fig. 1D). This effect was not influenced by the gelatin-coated plate system of culture, since we observed similar growth rate results when cells were cultured on MEF feeders (data not shown). These data suggest that our model iPSC line exhibits a strong proliferative response to serum and, depending on the media used, the presence of serum in iPSC cultures increases their propensity to differentiate, demonstrated by a reduction in self-renewal in GMEM plus serum conditions (Fig. 1B).

mTor has been reported to be a key effector regulating somatic cell proliferation [37], while ESCs from which mTor has been conditionally deleted fail to expand [29]. Given the clear difference in the proliferative capability of iPSCs compared to ESCs we investigated what effect treatment with the mTor inhibitor, rapamycin, would have. Rapamycin inhibited proliferation of both iPSCs and ESCs (Fig. 1C and D). Intriguingly, the proliferation rate of ESCs in KO SR medium after treatment with rapamycin was almost the same as that of untreated iPSCs (Fig. 1C) suggesting a putative impairment in mTor signaling in iPSCs. Immunoblotting to assess inhibition of mTor was carried out and as shown in Figure 1E, phosphorylation of S6 ribosomal protein, a downstream effector of the mTor-S6K1 pathway, was reduced upon addition of rapamycin, while phosphorylation of mTor itself was not. Interestingly, addition of serum to iPSCs led to an enhancement in S6 phosphorylation, which correlates with the enhanced proliferation of iPSCs in the presence of serum (Fig. 1C and D).

**Figure 2:**
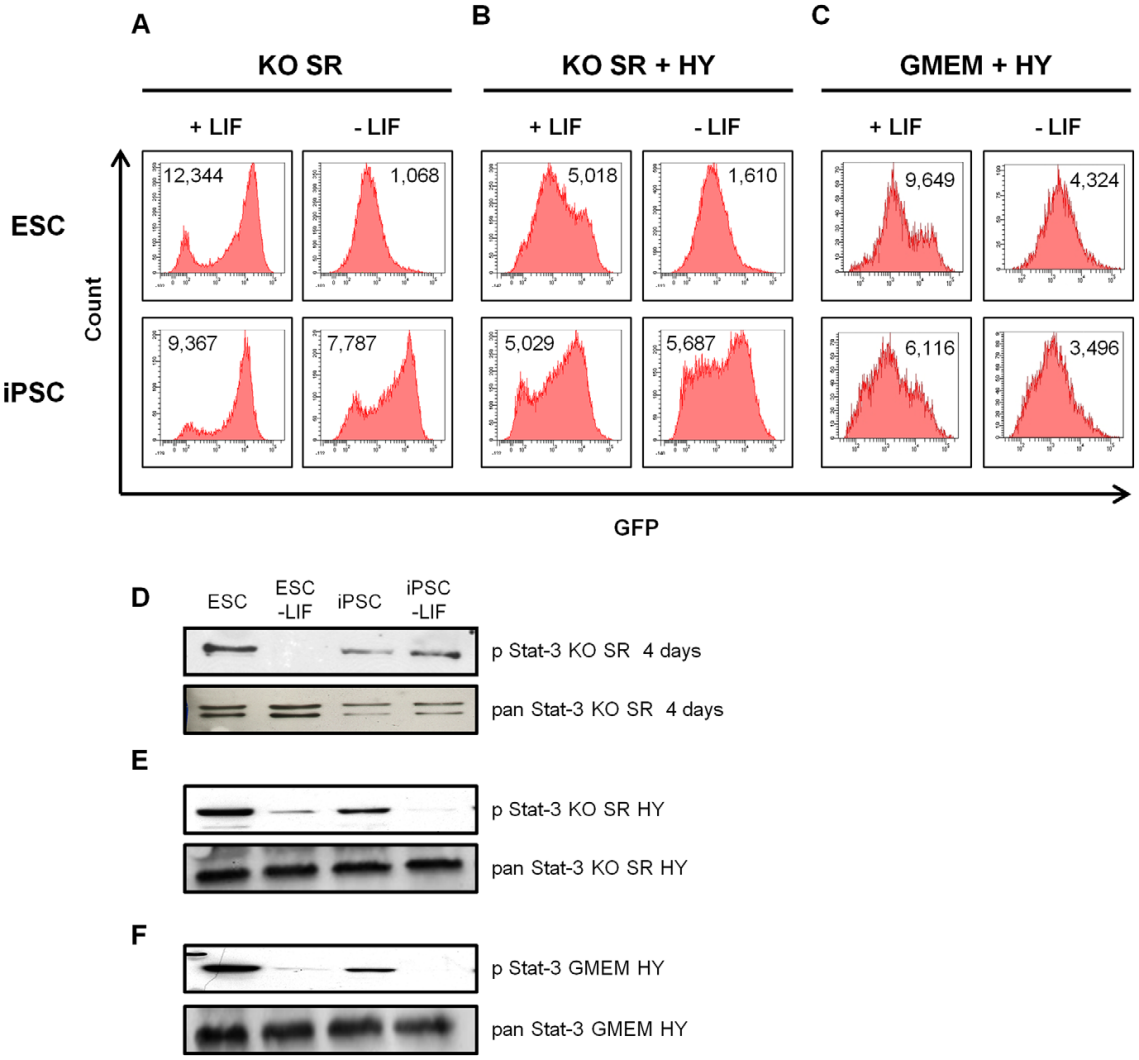
iPSCs are less sensitive to LIF withdrawal than ESCs. ESCs or iPSCs were plated at a concentration of 8000 cells/cm^2^, harvested after 4 days and analyzed for Nanog-GFP expression by flow cytometry. Cells were grown in KO SR (**A**), KO SR supplemented with 10% (v/v) Hyclone serum (HY) (**B**) or GMEM supplemented with HY (**C**) in presence or absence of LIF, as indicated. Numbers shown on the histograms are the mean of FL1 fluorescence intensity (MFI). One representative experiment out of four is shown. **D** ESCs or iPSCs were seeded at 8500 cells/cm^2^ in KO SR in the presence or absence of LIF. Protein extracts were prepared after 4 days incubation and immunoblotting performed with the antibodies indicated. Following detection of pY705 Stat-3, blots were stripped and reprobed to detect total levels of Stat-3. **E** and **F** Cells were seeded at 8500 cells/cm^2^ in KO SR plus 10% (v/v) HY (**E**) or GMEM supplemented with 10% (v/v) HY (**F**), in the presence or absence of LIF. After 48 h protein extracts were prepared and immunoblotting performed as indicated.

To further investigate the influence of culture environment on the fate of iPSCs, we made use of the fact that both the iPSC and ESC lines express GFP under the control of the endogenous Nanog promoter [3], [36]. Expression of Nanog has been reported to be heterogeneous in ESC cultures, with a low Nanog state making cells more susceptible to differentiation inducing cues and a high Nanog state being associated with maintenance of pluripotency [36]. The profiles of GFP expression, a read-out for Nanog expression, were analyzed by flow cytometry following culture of cells in different conditions. When cultured in KO SR and LIF (Fig. 2A +LIF panel), the profile of Nanog-GFP expression was similar for both iPSCs and ESCs, with the majority of cells having high GFP expression and a smaller proportion of cells exhibiting low levels of GFP expression, consistent with previous reports [36]. When serum was added (Fig. 2B +LIF panel), there was a shift towards more heterogeneous Nanog-GFP expression in ESCs such that the majority of cells in the population exhibited intermediate levels of GFP. In contrast, iPSCs maintained a larger proportion of cells expressing high levels of GFP. When cultured in GMEM supplemented with LIF and Hyclone serum (Fig. 2C +LIF panel), expression of the GFP reporter was again heterogeneous. The effect of removing LIF from the cultures on Nanog-GFP expression was also examined. Surprisingly, even after 4 days in KO SR medium in the absence of LIF, the majority of iPSCs retained high levels of GFP expression (Fig. 2A, -LIF panel), which was in stark contrast to the lower levels of GFP detected in ESCs under the same conditions. This effect was less pronounced when serum was also present in the KO medium (Fig. 2B -LIF panel) and GFP expression appeared to be more heterogeneous, although again expression was higher in iPSCs than ESCs. In GMEM plus serum both ESCs and iPSCs showed similar patterns of lowered GFP expression (Fig. 2C –LIF panel). These results suggested that the iPSCs are less sensitive to withdrawal of LIF than ESCs and so we decided to examine the status of Stat-3 phosphorylation, since it is a direct target of LIF-induced signaling. In iPSCs cultured in KO SR Stat-3 phosphorylation at Y705 was retained even after 4 days of LIF withdrawal, whereas phosphorylated Y705 Stat-3 was undetectable in ESCs in the same conditions (Fig. 2D). In KO SR plus serum (Fig. 2E) or GMEM plus serum (Fig. 2F), pY705 Stat-3 decreased following removal of LIF in both ESCs and iPSCs. Taken together with earlier results, these data suggest that the iPSC line is less sensitive to LIF withdrawal than is typical for mouse pluripotent ESC lines and retains characteristics of self-renewing cells, i.e., Nanog expression and Stat-3 Y705 phosphorylation, for extended periods in the absence of LIF.

### iPSCs exhibit reduced levels of Erk MAP kinase activation

**Figure 3:**
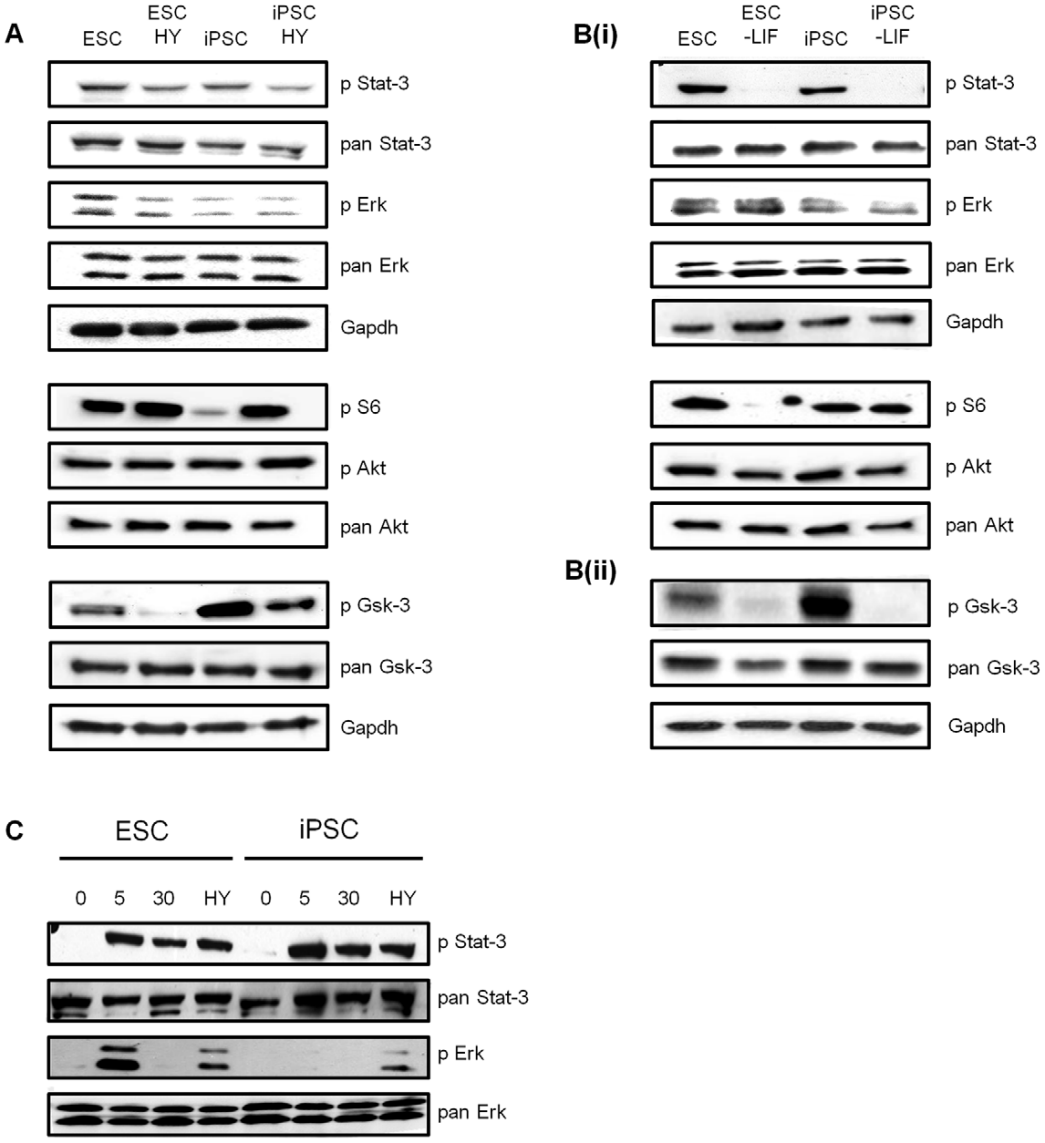
Expression and phosphorylation status of key signaling intermediates in ESCs and iPSCs. **A** and **B** ESCs or iPSCs were seeded 8000 cells/cm^2^ in KO SR plus LIF. After 24 hours, 10% (v/v) Hyclone serum (HY) was added where indicated (**A**), or cells were washed 3 times with PBS before LIF-containing or LIF-free KO SR was added to the cultures as indicated (**B**). After a further 24 hours incubation, protein extracts were prepared, separated through 10% acrylamide gels using SDS-PAGE and immunoblotted using the antibodies indicated. Blots in **A** and **B**(i) were all generated using cell extracts from one replicate and those in **B**(ii) from an independent experimental replicate. **C** ESCs or iPSCs were seeded at 8500 cells/cm^2^ in GMEM 10% (v/v) Hyclone serum (HY) plus LIF, then 24 h later washed and deprived of LIF and serum for 4 h. 5000 U/ml of LIF or 10% (v/v) HY were added and proteins extracted following 0, 5, 30 min treatment with LIF or 30 min treatment with serum. The phospho-proteins indicated were detected by immunoblotting of the same membrane, which had been cut referring to the size of the protein marker, then stripped and re-probed with the corresponding pan antibody. Results generated from the same blots are grouped, with each series terminating with the respective Gapdh as loading control.

Having established the basic growth characteristics of iPSCs, we next investigated whether the molecular signaling pathways known to be involved in regulating pluripotency of mouse ESCs are also involved in regulating the cell fate decisions of iPSCs. Initially we assessed the basal levels of activation of key pathways after cells had been cultured for 48 h in KO SR supplemented with LIF in the absence or presence of serum (Fig. 3A) or cultured in KO SR in the presence or absence of LIF (Fig. 3B). As shown in Figures 3A and 3B, the levels of protein expression for key signaling components, including Stat-3, Erks, Gsk-3 and Akt were similar in ESCs and iPSCs. In addition, the levels of phosphorylation of key activation motifs on Stat-3 (Y705) and Akt (S473) were similar in ESCs and iPSCs. However, we consistently observed that Erk1 and 2 exhibited reduced levels of phosphorylation in iPSCs, which was even more dramatic when cells were cultured in GMEM plus serum (Figure S1). In relation to phosphorylation of the S6 ribosomal protein at S235 and 236, in some experiments we observed lower levels of phosphorylation in iPSCs compared to ESCs in basal conditions (Fig. 3A and see below), but in other experiments no difference was observed (Fig. 3B(i); also see Fig. 1E and below). It is known that S6 phosphorylation is sensitive to the rate of cell proliferation, so this variability may be more a reflection of growth rates, with the slower rate of proliferation of iPSCs versus ESCs resulting in reduced S6 phosphorylation in some cases. However, we consistently observed enhanced levels of S6 phosphorylation when serum was included in iPSC culture medium (Fig. 1E and Fig. 3A). Interestingly, levels of S9/20 phosphorylation on Gsk-3 α/β isoforms were found to be consistently increased in iPSCs, compared to ESCs and declined in the presence of serum or upon LIF removal (Fig. 3A and B(ii)).

As we observed a reduction in levels of phosphorylated and active Erk1 and 2 in iPSCs, we examined the ability of acute stimulation with LIF (in the absence of serum and following 4 h LIF withdrawal) to induce phosphorylation and activation of Stat-3 and Erk1 and 2. As shown in Fig. 3C, short-term stimulation with LIF (5 and 30 mins) induced phosphorylation of Y705 of Stat-3 in both ESCs and iPSCs. A 30 min treatment with serum also induced Stat-3 phosphorylation at Y705. Interestingly, while a 5 min treatment with LIF induced a robust increase in Erk1 and 2 phosphorylation in ESCs, LIF appeared to be unable to activate Erk1 and 2 in iPSCs, despite similar overall levels of Erk expression in both cell types. Serum treatment served as a control and induced similar levels of Erk1 and 2 phosphorylation in ESCs and iPSCs. These data indicate that in the iPSC line used as our model, LIF receptor signaling is not as efficiently coupled to MAP kinase signaling as it is in pluripotent ESCs. However, this pathway is intact and presumably the basal phosphorylation measured in iPSCs arises due to the actions of other factors present, either produced by the iPSC cells themselves (ESCs produce Fgf4 which strongly induces Erk1 and 2 activation [38]) or present in the culture media.

### Perturbation of PI3K and Gsk-3-dependent signaling have opposing effects on the fate of iPSCs

Having established the signaling pathways that appear active in iPSCs, we then examined which of these played a role in controlling their fate using a series of small molecule inhibitors. ESCs and iPSCs were cultured for 4 days in the presence or absence of inhibitors (the doses of which had been optimized in previous studies [24], [25], [31]) and expression of GFP, as a read-out of Nanog expression, was assessed by flow cytometry.

**Figure 4:**
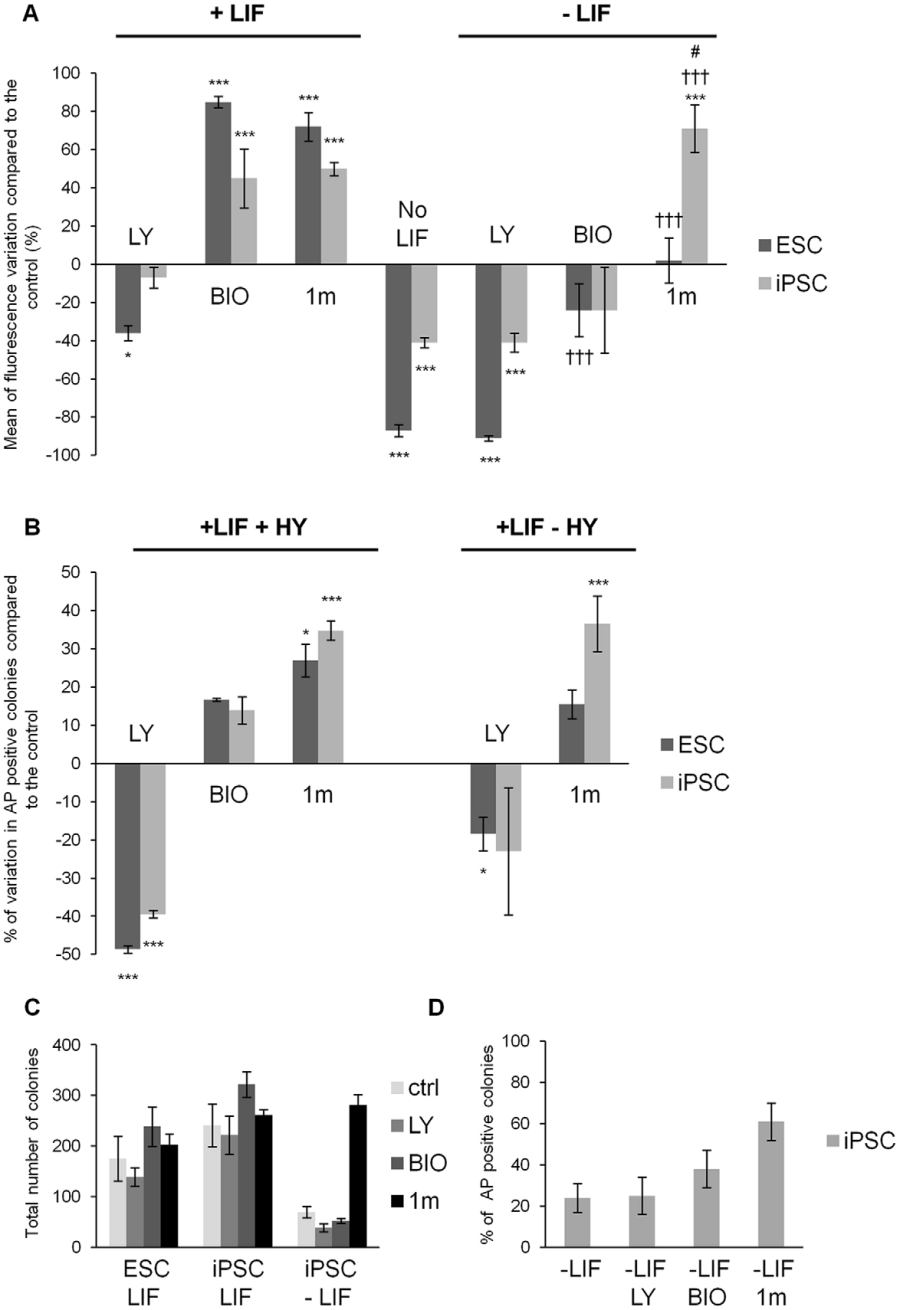
iPSCs demonstrate enhanced responsiveness to Gsk-3 inhibition. **A** ESCs and iPSCs were plated at a density of 8000cells/cm^2^ in KO SR plus or minus LIF as indicated. After 24 hours, inhibitors were added to the medium at the following concentrations: LY294002: 5 µM; BIO: 2 µM; 1 m: 2 µM; DMSO 1∶10000 as control, and cultured for 4 days. Following treatment, Nanog-GFP expression was assessed by flow cytometry and the mean of fluorescence intensity (MFI) of Nanog-GFP was calculated for each condition. The histogram bars represent the variation in Nanog-GFP MFI in treated samples, compared to the KO SR plus LIF (DMSO treated) control condition, expressed as a percentage. The mean of three independent experiments ± SEM are shown. Two-tailed paired t-tests indicate the following significance values: *** = p<0.005, * = p<0.05 are referred to the DMSO plus LIF condition; ††† = p<0.005 † = p<0.01 are referred to the DMSO minus LIF condition; # = p<0.01 is referred to the plus LIF 1 m-treated condition. **B** The percentage of alkaline phosphatase positive ESC or iPSC colonies generated in KO SR plus LIF in the presence or absence of 10% (v/v) Hyclone serum (HY) were measured. The data represent the percentage variation in alkaline phosphatase (AP) positive colonies compared with the DMSO treated control. The average values ± SEM are shown from three independent experiments. Two-tailed paired t-test: *** = p<0.005, * = p<0.05 are referred to the DMSO plus LIF. **C** iPSCs were cultured in KO SR without LIF and supplemented with 10% (v/v) HY. The total numbers of colonies generated are shown and are compared with the total number of colonies generated by ESCs and iPSCs in KO SR plus LIF plus serum. The percentage of alkaline phosphatase positive colonies are shown in histogram **D**.

To investigate the effect of PI3K signaling perturbation, we used LY294002 [39], a broad selectivity PI3K inhibitor which has been shown to lead to loss of ESC self-renewal [31]. As shown in Fig. 4A, in KO SR in the presence of LIF, inhibition of PI3K signaling with LY294002 led to a reduction in Nanog-GFP expression in ESCs, but not in iPSCs. In the absence of LIF, Nanog-GFP expression decreased in both ESCs and iPSCs, although the reduction was less for iPSCs, consistent with the data shown in Fig. 2B and the inclusion of LY294002 had no additional effect on either cell type. To analyze the effect of Gsk-3 inhibition, we used BIO, a commercially available Gsk-3 inhibitor that has been reported to enhance ESC self-renewal and Nanog expression [25] and 1 m, a Gsk-3 inhibitor that demonstrates greater selectivity than BIO and that also enhances self-renewal of mouse ESCs [24]. Figure 4A shows that inhibition of Gsk-3 with either BIO or 1 m led to a pronounced increase in GFP expression in both cell types, consistent with previous results [24], [25]. BIO had modest effects, only significant in ESCs, while 1 m prevented the decrease in GFP expression apparent in the control LIF-deprived ESCs (-LIF, Fig. 4A) and significantly elevated GFP expression in iPSCs.

To complement these analyses, we also assessed the effects of small molecule inhibitors on cell fate control of iPSCs using alkaline phosphatase staining as an alternate measure of self-renewal. Based on our earlier observations showing that self-renewal of iPSCs is best maintained in KO SR medium (Fig. 1), we carried out these studies in KO SR plus LIF in the presence or absence of serum. In the presence of LIF and serum, inhibition of PI3K signaling with LY294002 decreased the proportion of alkaline phosphatase positive, self-renewing iPSC and ESC colonies (Fig. 4B). In the absence of serum inhibition of PI3Ks had no significant effect on iPSC self-renewal (+LIF –HY, Fig. 4B). These results, along with those shown in Fig. 4A, suggest that whereas inhibition of PI3K signaling in ESCs leads to loss of self-renewal, as reported previously [28], [31], under the different conditions tested, self-renewal of iPSCs is only perturbed by LY294002 treatment in the presence of serum. This may be related to the requirement for factors contained in serum to maintain cells as they differentiate. In contrast, incubation with Gsk-3 inhibitors in the presence of LIF and serum increased the proportion of alkaline phosphatase positive colonies (Fig. 4B). In this case the absence of serum didn't change the effect in a significant way (+LIF –HY, Fig. 4B), as addition of 1 m enhanced the proportion of alkaline phosphatase positive colonies present over the levels in DMSO-treated controls by approximately 40% in each case.

Based on our finding that 1 m treatment maintains Nanog-GFP expression in iPSCs following LIF removal, we also tested whether iPSCs could generate alkaline phosphatase positive colonies in the absence of LIF. While ESCs formed very few colonies without LIF in KO SR plus serum (data not shown), iPSCs did generate colonies, albeit in reduced numbers compared to in the presence of LIF (Fig. 4C). Importantly, the inclusion of 1 m to iPSCs cultured in KO SR plus serum without LIF led to the formation of comparable proportions of alkaline phosphatase positive colonies (Fig. 4D) to those formed in the presence of LIF (Fig. 1B), as well as similar numbers of colonies (Fig. 4C). These results are consistent with our earlier data demonstrating decreased sensitivity of iPSCs to LIF withdrawal (Fig. 2A and B, -LIF panel). Thus, in the absence of LIF, inhibition of Gsk-3 in iPSCs not only maintains Nanog-GFP expression (Fig. 4A), but also enhances self-renewal (Fig. 4D) and survival (Fig. 4C) suggesting modulation of Gsk-3 activity is key to determining the fate of iPSCs.

**Figure 5:**
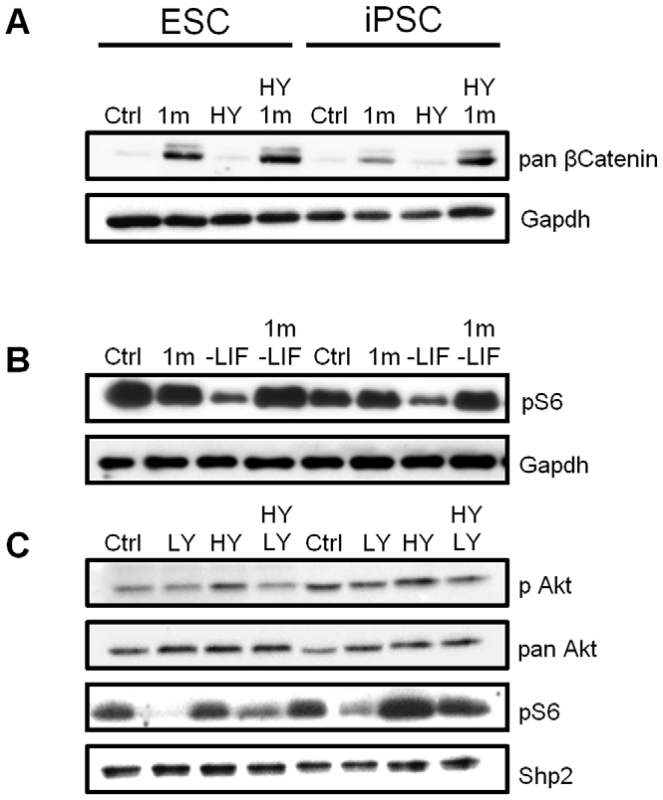
Effect of perturbation of key self-renewal signaling pathways in ESCs and iPSCs. ESCs and iPSCs were seeded in KO SR plus or minus LIF as indicated. After 24 hours 2 µM 1 m and 10% (v/v) Hyclone serum (HY) alone or together (**A**), 2 µM 1 m (**B**), 5 µM LY294002 and 10% (v/v) HY (**C**) or DMSO 1∶10000 (in all controls), were added. After a further 24 hours proteins were extracted and immunoblotting performed as indicated. Blots were stripped and re-probed with Gapdh, pan Akt or Shp2 antibodies to assess loading.

In light of the results of our functional analyses, we next assessed the effects that treatment of ESCs and iPSCs with these small molecule inhibitors had on the activity of key signaling pathways. As the Gsk-3 inhibitor BIO was shown to have similar effects to the 1 m molecule, but is known to be less selective [24], only 1 m was used for the following experiments. Given the ability of Gsk-3 inhibition to promote maintenance of iPSC self-renewal in both the presence and more particularly the absence of serum, we were initially most interested to examine the consequences of Gsk-3 inhibition on signaling in iPSCs. In the presence of LIF or LIF and serum, inhibition of Gsk-3 with 1 m for 24 hours resulted in significant elevation of β-catenin levels in both ESCs and iPSCs, consistent with stabilization of β-catenin protein (Fig. 5A). Interestingly, basal levels of S6 phosphorylation declined upon LIF withdrawal in both ESCs and iPSCs. However, inclusion of 1 m maintained levels of pS6 phosphorylation in ESCs and iPSCs cultured without LIF (Fig. 5B), which could contribute to enhanced survival. In ESCs, when LY294002 was used to inhibit PI3K signaling, a decline in phosphorylation of Ser 473 of Akt, a key downstream target of PI3K signaling, was only readily detectable in the presence of serum (Fig. 5C), although phosphorylation of S6 was clearly reduced by LY294002 treatment in both the absence and presence of serum. Interestingly, in iPSCs, LY294002 had less effect on S6 phosphorylation in the presence of serum, compared to a dramatic reduction in the absence of serum, consistent with increased responsiveness of this iPSC line to serum. Taken together, these data suggest that Gsk-3 plays a predominant role in regulating the fate of iPSCs and that S6 phosphorylation is particularly sensitive to presence of serum.

### iPSCs change in morphology and behavior following long-term culture

**Figure 6:**
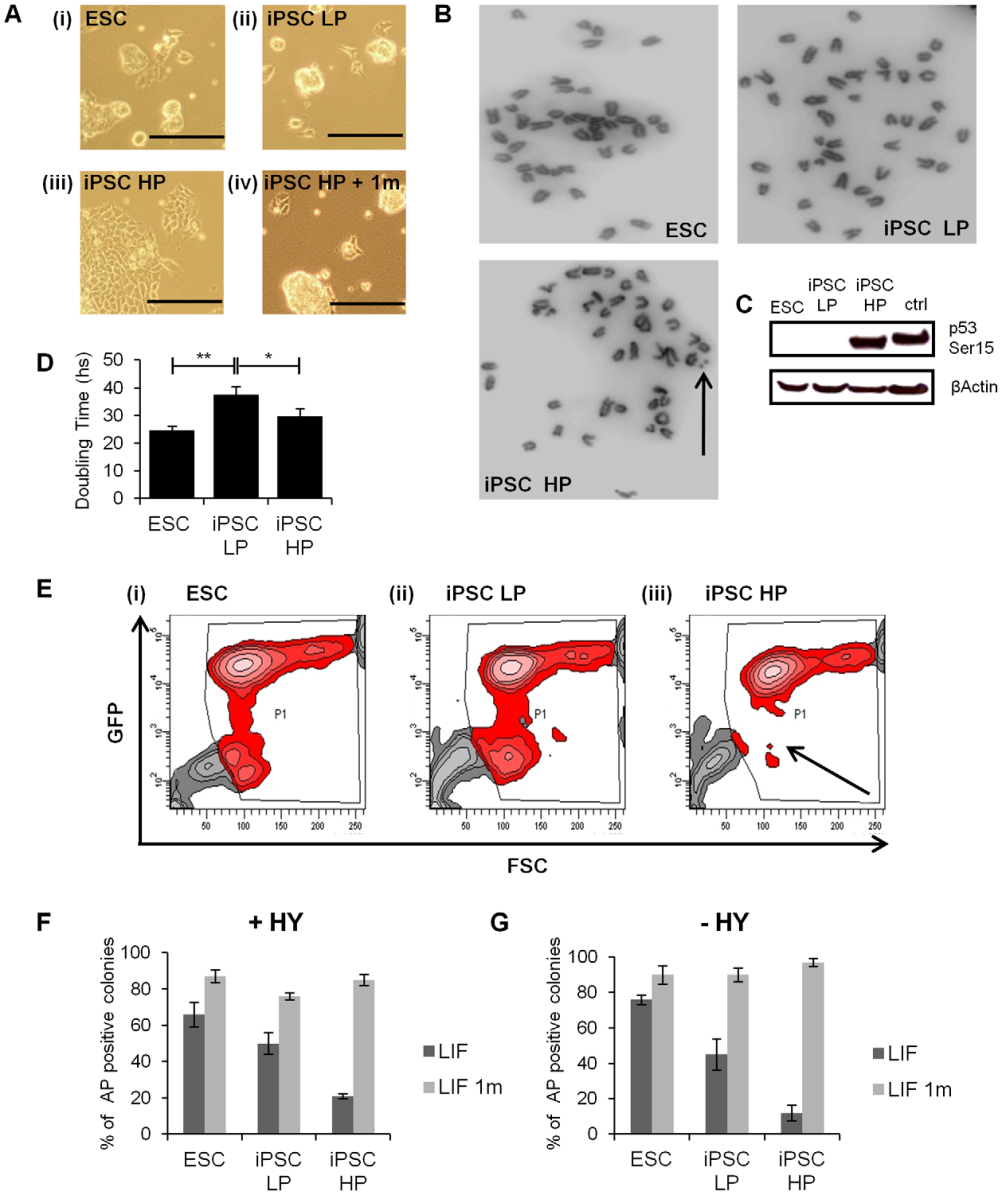
High passage iPSCs exhibit altered morphology and response to stimuli. Cells were plated at a density of 8500 cells/cm^2^ in KO SR plus LIF. **A** iPSCs changed morphology after several passages (iii), compared with ESC (i) or iPSC low passage (ii), but reinstate an ESC-like morphology after 24 h treatment with 2 µM 1 m (iv). iPSCs were considered as low passage (iPSC LP) with passages <25 and high passage (iPSC HP) with passages >40. **B** Chromosome spreads of ESCs and low/high passage iPSCs. The arrow indicates chromosome aberrations in iPSC HP. One representative karyotype out of 20 from each cell line is shown. **C** 4 days after seeding, ESC or iPSC proteins were extracted, separated through 10% acrylamide gels using SDS-PAGE and immunoblotted using phospho-S15 p53 antibody. β-actin antibody was used as loading control. As a positive control, protein extracted from irradiated T lymphocytes was used. **D** Cells were plated at day 0 and then harvested after 48 (D2), 72 (D3) and 96 (D4) hours. Cells were counted in triplicate. The average doubling times ± SEM are shown from two independent experiments. Doubling times were calculated using free software available from www.doubling-time.com. Two-tailed paired t test: ** = p<0.01 * = p<0.05. **E** iPSCs low/high passage or ESCs were harvested 4 days after seeding and Nanog-GFP expression analyzed by flow cytometry. Contour plot graphs are shown and the missing Nanog-GFP low population in iPSC HP indicated by an arrow. **F** and **G** Alkaline phosphatase positive colonies were counted after growing in KO SR plus LIF supplemented with (**F**) or without 10% (v/v) Hyclone serum (HY, **G**). After 24 hours, 2 µM 1 m was added and kept in the medium until the cells were fixed and stained. The average values ±SEM are shown from three independent experiments.

During the course of culturing iPSCs we observed that between passage 25 and 40 their morphology began to alter, even when maintained in KO SR supplemented with LIF (Fig. 6A panel (iii)). The change of morphology was just one of the several differences apparent between iPSCs at low passage (iPSC LP, considered as passages <25) compared with the high passage iPSCs (iPSC HP, considered as >40 passages). First of all, iPSC HP showed genetic instability, with an abnormal karyotype and chromosomal aberrations (Fig. 6B and Table S1). This observation was reinforced by the observation that phosphorylation of Ser 15 of p53 was dramatically increased in iPSC HP (Fig. 6C), which is linked with genetic instability. Moreover, iPSC HP were found to proliferate at a rate comparable with that of ESCs (Fig. 6D), raising the possibility that these cells had undergone some form of adaptation. In spite of these changes, some characteristics remained stem cell-like, such as a high level of expression of the Nanog-GFP, even though the GFP negative population (indicated by the arrow in Fig. 6E panel (iii)) is lost in iPSCs HP. When the ability of iPSC HP to self-renew was tested in clonal assays, a lower proportion of alkaline phosphatase positive colonies were generated in KO SR plus LIF, in both the presence (Fig. 6F) and absence of serum (Fig. 6G). However, in the presence of 1 m, the percentage of alkaline phosphatase positive colonies increased dramatically (Fig. 6F and G). This effect, coupled with a reinstatement of an ESC-like morphology in the presence of 1 m (Fig. 6A panel (iv)), suggests that high passage iPSC HP, like their low passage predecessors, are highly sensitive to Gsk-3-dependent signaling. Finally, iPSC HP were not able to grow in self-renewal assays without LIF (data not shown), suggesting a recovery in LIF dependence.

## Discussion

In this study we undertook a comparison of the molecular signaling pathways that contribute to maintenance of the stem cell state of mouse iPSCs and ESCs. Our studies demonstrate that the iPSC line we used as our model exhibits a significant response to factors contained within serum, accompanied by reduced sensitivity to the withdrawal of LIF. While basal levels of expression and phosphorylation of components of a number of signaling pathways known to regulate self-renewal of mouse ESCs were similar in iPSCs and ESCs, our studies revealed a key role for Gsk-3 in controlling iPSC fate. Upon LIF withdrawal, inhibition of Gsk-3 was able to maintain iPSCs in a self-renewing state for short periods, a response not observed with ESCs. Furthermore, inhibition of Gsk-3 improved survival of iPSCs in the absence of LIF and also increased the ability of high passage iPSCs to self-renew. High passage iPSCs exhibited evidence of genomic instability and altered responsiveness. Together our findings highlight the importance of characterizing not only the molecular signatures of reprogrammed iPSCs, but also their responses to the external environment and signals controlling their behavior.

The equivalency between iPSC lines and ESC lines is still somewhat controversial. Many reports indicate that there is a high degree of similarity between the cell types [3], [7], [9], [40], [41], but more recently important differences have also been reported [16], [17], [42]–[44]. Although we have only examined the responses of one iPSC line in our studies, meaning we cannot exclude the possibility that our data reflect features exclusive to this particular line, our data are consistent with the idea proposed that iPSC lines retain a ‘memory’ of their cell of origin. Several recent high profile publications have demonstrated that iPSC lines can retain both genetic and epigenetic ‘memories’ of the differentiated cell from which they were derived [16], [17], [42], [43]. In addition, comparison of human ESC and iPSC line proteomes and phosphoproteomes has shown there are reproducible differences which are consistent with iPSC lines retaining residual regulatory properties characteristic of their somatic cell of origin [45]. Our study suggests that this may also translate into the response of iPSCs to external signals. The iPSC line we used as our model was derived from primary mouse fibroblasts [3], which require serum to support their proliferation. It was interesting to find, therefore, that this iPSC line displayed a dramatic response to serum. In the absence of serum, iPSCs grew significantly slower than ESCs. However, when supplemented with serum, iPSC proliferation exceeded that of ESCs and this was accompanied by an increase in phosphorylation of the S6 ribosomal protein, consistent with enhanced cell metabolism/proliferation. We propose that a useful additional measure to assess the completeness of reprogramming may be to investigate if iPSC lines retain any response to factors that normally act upon the differentiated cells from which they were derived. We consider this important as residual responsiveness to such stimuli could also be retained in differentiated progeny derived from iPSCs, which could lead to aberrant cellular behaviors, highly undesirable in a cell-transplantation setting.

In addition to exhibiting responsiveness to serum-containing factors, our analyses reveal that our model iPSC line shows diminished sensitivity to withdrawal of LIF. Normally, mouse iPSCs require LIF for their self-renewal, although at least two examples of LIF-independent IPSC lines have been reported. Whether the reprogramming conditions used, epiblast stem cell conditions [14] or presence of a human fibroblast feeder layer [15], influenced the generation of these LIF-independent iPSCs is currently unclear. In KO SR in the presence or absence of serum, iPSCs retained higher levels of Nanog-GFP reporter expression when LIF was removed compared to ESCs, which rapidly lost GFP expression (Fig. 2A&B). This characteristic of iPSCs could be linked to the fact that they proliferate more slowly, meaning that they may also differentiate more slowly. However, we do not think this is the case because in self-renewal assays, iPSCs formed colonies in the absence of LIF (Fig. 4C), whereas very few ESC colonies survived under the same conditions. It also important to note that although Stat-3 phosphorylation in basal conditions and in short-term LIF stimulations followed similar kinetics to those of ESCs, in extended cultures, Stat-3 phosphorylation could be detected in iPSCs after several days of culture without LIF. Under the same conditions, Nanog-GFP was also retained in a high proportion of cells, consistent with iPSCs maintaining features of a pluripotent cell state for a period of time following withdrawal of LIF.

Several of the signaling pathway components we examined as part of this study were expressed and phosphorylated at similar levels in iPSCs and ESCs under basal conditions (KO SR plus or minus serum in the presence of LIF) including Stat-3, Akt and mTor. Therefore, it was interesting that consistent differences were observed in phosphorylation of Erk1, Erk2 and Gsk-3. The MAPK pathway is involved in cell proliferation, survival and differentiation [46]. Unexpectedly, in the iPSCs we used for the majority of these studies (at between 19 and 24 passages) acute stimulation with LIF was unable to induce detectable phosphorylation of Erk1 and Erk2. The reason for this un-responsiveness to LIF is currently unclear, however, the pathway appears intact as serum was shown to induce Erk1 and Erk2 phosphorylation in short-term treatments. Gsk-3 has been implicated in the regulation of mouse ESC self-renewal by a number of groups, including our own [23]–[25]. It has also been reported that inhibition of Gsk-3 can accelerate the full reprogramming of ‘pre iPSC’ [47]. Therefore, it was of considerable interest to discover that our model iPSC line demonstrated elevated levels of Gsk-3 phosphorylation compared to ESCs and the fate of this iPSC line was particularly sensitivity to inhibition of Gsk-3 activity. For example, treatment with the Gsk-3 inhibitor 1 m was able to promote maintenance of Nanog-GFP expression in both ESCs and iPSCs in the presence of LIF, but also, most notably for iPSCs, in the absence of LIF. Treatment of iPSCs with 1 m was also able to promote cell survival and support formation of alkaline phosphatase positive colonies in the absence of LIF. It has been demonstrated that when activation of Erk MAP kinases is diminished, down-modulation of Gsk-3 becomes crucial to maintenance of pluripotency [23], which reflects the situation we observe in this iPSC line.

Another key aspect of our study was the finding that the characteristics of our iPSC model line changed with increasing passage. There are a number of possible explanations that could account for these observations; this line may be genetically unstable, it may have adapted to the tissue culture conditions used or the iPSC line could have regressed to a more differentiated state. In support of the first possibility are our data demonstrating that at higher passage numbers (greater than 40) this iPSC line exhibited karyotypic and chromosomal abnormalities. Furthermore, a change of morphology of iPSCs was consistently observed when they reached passage 40 or more and this was not stochastic. The high phosphorylation state observed for p53 in HP iPSC is also consistent with genetic instability, although p53 is known to be involved in many biological responses, not just in response to DNA damage. In previous studies iPSC lines have been demonstrated to be genetically stable [1], [7], [48], [49], although it is worth noting that cells at lower passage numbers were investigated in these cases, compared with our present work. Regarding “regression” to a more differentiated state, it is quite common during iPSC generation to observe colonies that are not completely undifferentiated, but share some markers and characteristics with pluripotent stem cells, although they fail the common pluripotency test [50]. Such cells have been referred to as “pre iPSC cells”. So, a partial but not complete differentiation could occur in iPSCs during long-term passage and in support of this is the fact that the proportion of self-renewing cells was considerably lower in iPSC HP compared to iPSC LP. This was somewhat surprising in view of the fact that iPSC HP have still very high Nanog GFP expression. Interestingly, we demonstrate that inhibition of Gsk-3 in iPSC HP restores their ability to generate self-renewing colonies and cells treated in this way revert to a morphology that more closely resembles low passage iPSCs and ESCs. This response is very reminiscent of the effects of Gsk-3 inhibition reported by Silva et al., [47], where they demonstrated that inhibition of Gsk-3 accelerated the reprogramming of pre iPSC to a fully reprogrammed state.

Taken together our data show an iPSC cell line, even if considered stable and respecting all the stringent parameters of pluripotency, can have important differences in response to external stimuli and regulation of self-renewal compared with ESCs. It has been shown that iPSCs are very sensitive to the environmental conditions during the reprogramming [13]–[15] and they can also adapt to their culture conditions [17]. Here we confirm and extend these findings. Although our results contrast with some previous reports, and we have used only one line for our studies, our findings do raise an important general issue for iPSC biology, especially for those who want to use iPSCs for applications such as drug discovery/screening and cell-based therapies. Further more extensive analyses are required to determine if the characteristics described here are commonly observed with other iPSC lines. Should such differences be more generally observed it will be very interesting to link them with different tissues of origin and possibly also the reprogramming technique. As the field of iPSC biology progresses it is becoming increasingly clear that establishing strict parameters for evaluating full reprogramming and stability will be vital to ensuring safety and efficacy.

## Materials and Methods

### ESC and iPSC cell culture

The culture of mouse ESCs and iPSCs were carried out as described previously [31], [51], [52]. The E14tg2a ESC line [36] and the iPSC line [3] (less than 25 passages from derivation) were used throughout this study. Culture media used were KnockOut-DMEM (GIBCO) supplemented with 15% (v/v) KO Serum Replacement (GIBCO) and 1000 units\ml of LIF; GMEM (GIBCO) supplemented with 10% (v/v) Hyclone FBS and 1000 units/ml LIF; KnockOut-DMEM supplemented with 15% (v/v) KO Serum Replacement, 10% (v/v) Hyclone serum and 1000 units/ml LIF.

### Cell proliferation

Cells were cultured on 24-well trays coated with gelatin at a starting concentration of 24000 cells/cm^2^ for the KO SR condition and 8000 cells/cm^2^ for the KO SR HY condition. At each time point cells were harvested, stained with trypan-blue and viable versus non-viable cells counted in triplicate using a Neubauer chamber. Doubling times were calculated by the free-software available from the web site www.doubling-time.com.

### Alkaline phosphatase staining

To assess self-renewal, ESCs or iPSCs were plated at 1500 cells per gelatin-coated well of a six well tray and cultured either without LIF or with the addition of 1000 units/ml of LIF. 6 hours after plating (to permit adherence) inhibitors (5 µM LY294002, 2 µM BIO, 2 µM 1 m or 1∶10000 DMSO as control) were added without medium change. To detect colonies expressing the alkaline phosphatase enzyme, a marker of pluripotency, after 5 days of culture cells were washed in PBS, fixed in 100% methanol and stained with Fast Violet salt (Sigma) dissolved in 0.1 M Tris pH 9.2, containing 200 mg/ml Naphtol AS-MX phosphate. Alkaline phosphatase-positive colonies were counted in triplicate for each experiment using an Olympus XI51 inverted microscope.

### Protein extraction and immunoblotting

Cell extracts were prepared, protein concentrations determined and 20 µg of protein separated by SDS-PAGE and transferred to nitrocellulose as previously described [53]. Immunoblotting was carried out with the following primary rabbit polyclonal antibodies: 1∶1000 for rabbit polyclonal antibodies recognizing dual phosphorylation of Erk1 and 2 at Thr202/Tyr204 (anti-pErk, Cell Signaling Technology 9101), phospho-tyrosine 705 of Stat-3 (anti-pSTAT-3, CST 9131), phospho-serine 473 of Akt (anti-pAkt, CST 9271), phospho-serines 21 or 9 of Gsk-3 α/β (anti-pGsk-3 α/β, CST 9331), phospho-serines 235/236 of S6 (anti-pS6 CST 4856), phospho-serines 411/418 of p70 S6 kinase (anti- p70S6 kinase CST 9204), phospho-serine 2448 of mTOR (anti-pmTOR CST 2976), phospho-serine 15 of p53 (anti p53 CST 9284), anti-pan β-catenin (CST 9562), anti-pan Akt (CST 9272); 1∶2000 anti-pan Erk (panErk, Santa Cruz Biotechnology, sc-93), anti-pan STAT-3 (panStat-3, sc-482), anti-pan SHP-2 (sc 293), anti-pan mTOR (CST 2983) anti-pan p70S6 kinase (CST 9202) anti β-Actin (SIGMA A5316) and anti GAPDH (CST 2118). An anti-rabbit secondary antibody conjugated to horseradish peroxidase (DAKO) was used for detection and blots were developed using ECL according to the manufacturer's directions (GE Healthcare). Protein relative quantification was carried out using an ImageQuant™ RT-ECL imager and analyzed using ImageQuant™ TL software (GE Healthcare). Blots were stripped and reprobed as previously described [53].

### Flow cytometry

ESCs and iPSCs were trypsinised, washed and resuspended in PBS containing 5% (v/v) FCS, 0.1% (w/v) sodium azide. Flow cytometry was carried out using a FACSCanto instrument (Becton Dickinson). Dead cells were excluded from the analysis based on forward and side scatter parameters. Mean fluorescence intensity (MFI) of the GFP reporter in the viable cell population of each cell sample was determined using FACSDiva software. The variation between MFI values of treated samples compared to control samples were calculated and expressed in percentage terms as either increases or decreases. The average difference in MFI values between samples from three independent experiments were determined and data presented on histograms.

### Karyotyping

Cells were seeded 8000/cm^2^ 24 h before treatment, then 0.1 µg/ml Colcemid (Sigma) was added into the medium and cells were incubated for 4 h at 37°C. Cells were then trypsinized, incubated for 10 min in 0.075 M KCl solution and then fixed with 3∶1 methanol: glacial acetic acid. Fixed cells were then dropped on to a cooled glass slide from a height approximately 1 meter, air dried and DAPI stained. The karyotypes of 20 independent cell spreads from each cell line were counted (Table S1).

## Supporting Information

Figure S1 **ESCs and iPSCs were plated at a density of 8500 cells/cm^2^ in GMEM plus 10% (v/v) Hyclone serum plus or minus LIF as indicated.** After 48 h proteins were extracted and immunoblotting performed with the indicated antibodies.
(TIF)

Table S1 **After chromosome spreading and DAPI staining, 20 single nuclei fields were counted for each cell line.** The different chromosome counts were expressed as a percentage and shown in the table.
(TIF)
