# Supplementary figures and images for: Differential Coupling of Self-Renewal Signaling Pathways in Murine Induced Pluripotent Stem Cells
Source: PLoS One. 2012 Jan 23;7(1):e30234. doi: 10.1371/journal.pone.0030234 (PMC3264619; doi:10.1371/journal.pone.0030234)

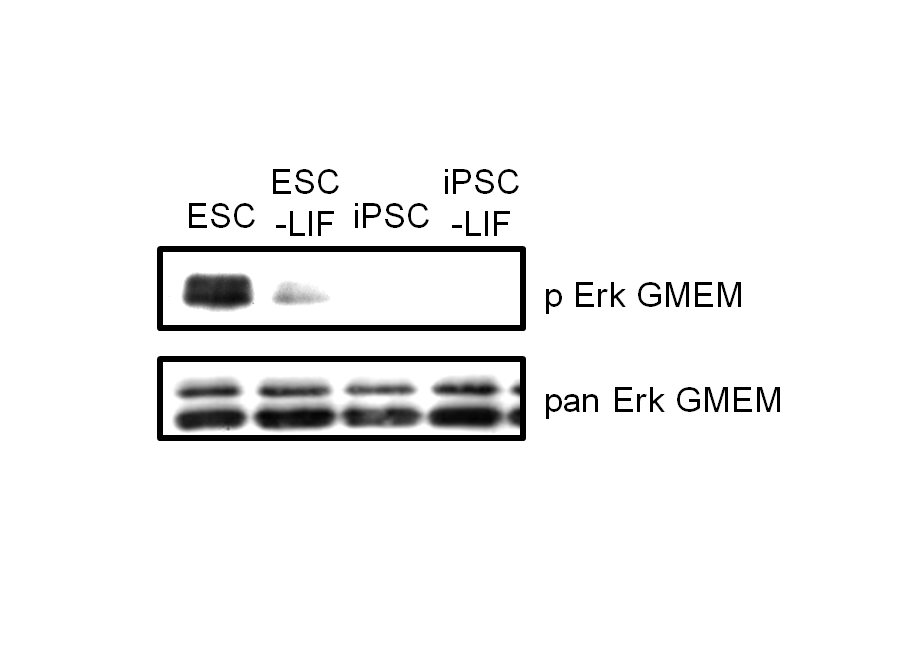

Supplement: Figure S1 — ESCs and iPSCs were plated at a density of 8500 cells/cm2 in GMEM plus 10% (v/v) Hyclone serum plus or minus LIF as indicated. After 48 h proteins were extracted and immunoblotting performed with the indicated antibodies. (TIF) [file pone.0030234.s001.tif]

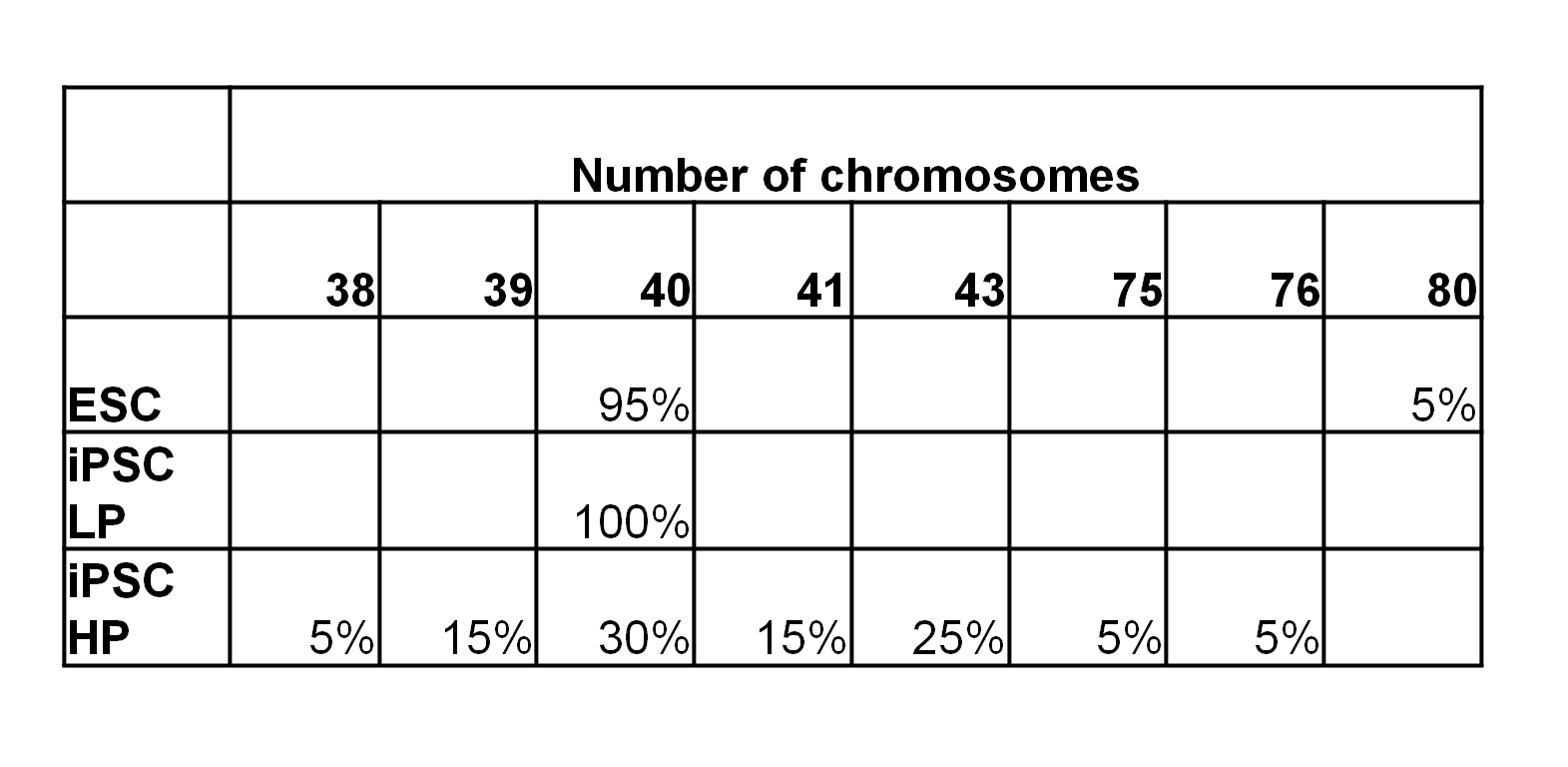

Supplement: Table S1 — After chromosome spreading and DAPI staining, 20 single nuclei fields were counted for each cell line. The different chromosome counts were expressed as a percentage and shown in the table. (TIF) [file pone.0030234.s002.tif]
